# Health-related quality of life and its determinants in patients with different dermatological disorders at the University of Gondar Comprehensive Specialized Hospital

**DOI:** 10.1186/s13104-023-06442-8

**Published:** 2023-08-31

**Authors:** Eyayaw Ashete Belachew, Ashenafi Kibret Sendekie

**Affiliations:** Department of Clinical Pharmacy, School of Pharmacy, College of Medicine and Health Sciences, University of Gondar, P.O. Box: 196, Gondar, Ethiopia

**Keywords:** Dermatology, Health-related quality of life, Skin disorders, Ethiopia

## Abstract

**Objectives:**

Evidence shows that majority of dermatological disorders affect the health-related quality of life (HRQoL) of patients. However, the extent of its negative impact and predictors has not been studied in Ethiopia. Thus, this study looked at assessing the HRQoL and determinants in patients with dermatological disorders (DDs) attending the University of Gondar Comprehensive Specialized Hospital (UoGCSH).

**Results:**

Patients with dermatological disorders (n = 400) were included in the final analysis using a systematic random sampling technique. The mean age of the participants was 39.79 (± 17.17) years. The average (± SD) score of EQ-5D-5 L was 1.92 (± 0.74). Regarding domains, pain/discomfort accounted for a higher proportion 59 (22.3%) followed by anxiety/depression 61 (15.3%). Receiving topical preparations (β = -0.399, 95% CI: -0.6, − 0.19; < 0.001), systemic only medication (β = -0.378, 95% CI: -0.607, -0.149; p = 0.002), having slight, mild, and moderate skin diseases found to have an inverse association with impaired HRQoL, (β = -0.654, 95% CI; -1.01, -0.290); p < 0.001), (β = -0.748, 95% CI: -0.960, -0.538; p < 0.001), and (β = -0.465, 95% CI: -0.642, -0.283; p < 0.001), respectively. Furthermore, age (β = 0.011, 95% CI: 0.006, 0.016; p = 0.001), long duration with skin disease (β = 0.046, 95% CI: 0.015, 0.352; p = 0.013), and presence of comorbidity (β = 0.251, 95% CI: 0.096, 0.402; p = 0.002) were significant predictors of HRQoL among dermatological disease patients.

**Conclusion:**

Patients with dermatological disease were found to have a compromised HRQoL. Pain /discomfort problems accounted for a higher proportion compared with other domains. Socio-demographic, clinical and medication-related variables were significantly associated with HRQoL.

## Introduction

Dermatological disorders (DDs) have a significant global public health impact, among the top 10 global diseases [1–3]. The most common prevalent skin disorders include acne, atopic dermatitis (eczema), psoriasis, rosacea, skin cancers, vitiligo, herpes zoster, sunburn, tinea pedis, melasma, and contact dermatitis [3].

Most of DDs are chronic and significantly reduce HRQoL among its patients [4]. This disorders are the fourth leading cause of non-fatal diseases, accounting for approximately 1.79% of total disease in the 2013 global burden report [5]. If DDs are not treated properly, several complications can occur [6]. In studies conducted in the United Kingdom [4] and Malaysia [7], the major complications of DDs and their impact on the physical, social, and psychological aspects of a patient’s HRQoL were reported. To accurately determine the impact of burden of skin disease, the patients’ HRQoL must also be considered [8].

Skin diseases have a negative impact on HRQoL. Various studies reported a negative impact of skin disorders including psoriasis, acne vulgaris, cutaneous lupus erythematous, alopecia areata, urticaria, and vitiligo on HRQoL [9–14]. A number of variables have been linked to the HRQoL of patients with a specific skin condition. For example, psoriasis patients’ HRQoL is associated with their age, employment status, marital status, disease duration, and self-reported severity [14]. The HRQoL of patients with cutaneous lupus erythematous is influenced by their gender, age, generalized disease, distribution of lesions, and severity [10]. Another study also disclosed that a correlation between low HRQoL and sex, education, income, the presence of systemic lupus erythematous, and disease severity of patients living with DDs [13]. Moreover, socioeconomic factors including large joint families, use of impure water, low education status and exchange of footwear with patients are the major contributing factors. The incidence rate of skin disorders is high due to these factors in resource-limited countries [5].

Despite the fact that they are classified as chronic diseases and among the top four most common diseases affecting the world population [15], the impact of skin disorders is widely underestimated. The direct and indirect cost of treating skin diseases in the United States was 75 and 11 billion US dollars (USD), respectively [5].

Dermatological disorders such as vitiligo and psoriasis affect majority of people of all races. Extensive affected skin may result in social withdrawal, low personal relationships, embarrassment in sexual function, stigmatization, distress, anxiety, and difficulty finding work, all of which can lead to depression and quality of life impairments. Despite significant dermatologic complications and their association with poor HRQoL, no study has used an EQ-5D-5 L to assess health-related quality of life and associated factors in patients with DDs in Ethiopia. The European Quality of Life Group developed the EQ-5D-5 L, a generic, multi-attribute, utility-based health status tool that has recently been advocated to assess patients’ self-reported experiences and perceptions about their health status [16]. Mobility, self-care, daily activities, pain or discomfort, and anxiety or depressive symptoms are the five components of this tool [17]. As a result, this is the first study to use the EQ-5D-5 L to assess HRQoL in patients with DDs. Furthermore, a better understanding of these variables and the establishment of a utility value may improve intervention design and evaluation, as well as the integration of HRQoLassessment during patient care and decision-making.

HRQoL measurements in patients with DDs are extremely important. Besides evaluating treatment plans, dermatologists can learn more about the psychosocial difficulties of skin-related illness patients by assessing HRQoL. Additionally, HRQoL evaluation can highlight the need for psychosocial and psychotherapy interventions in a specific patient [18]. In the vast majority of cases, DDs do not pose a life-threatening risk but greatly impact the patient’s emotional status, social relationships, and daily activities. Many times, assessing the impact of a disease differs between the patient and doctor, which can interfere directly with disorder management. The analysis of HRQoL questionnaire responses allows establishing a relationship between the disease and its overall impact on patient’s life, resulting in satisfactory analysis of disease indications and treatment outcomes [19]. Since there is a lack of information on the level of HRQoand its determinants among dermatological disordered patients in Ethiopia using EQ-5D-5 L instrument, therefore, this study aimed to assess HRQoL and its determinants among different skin disease patients at Gondar University Hospital in Ethiopia.

## Methods and materials

### Study setting and study period

A facility-based cross-sectional study was conducted at the UoGCSH dermatologic clinic from June to August 2022. The hospital is in the Amhara Regional State of Gondar and is located approximately 750 km from Addis Ababa, Ethiopia’s capital city. It is one of the country’s largest teaching hospitals, with a catchment area of approximately 7 million people.

### Study participants and sampling procedures

The study included patients aged 18 and above with any dermatologic disorder and a disease duration of more than or equal to six months. However, patients who had a history of mental illness or were physically unable to complete the survey were excluded.

### Sample size and sampling procedure

Because no studies used the EQ-5D-5 L in a similar study in Ethiopia, the sample size was determined using the single population proportion formula. To obtain a maximum sample size, an estimated proportion of patients with utility values above the average were deemed 50% and the sample size was calculated. $$n=\frac{{\left(\text{z}\frac{{\alpha }}{2}\right)}^{2}\text{p}(1-\text{p})}{{\text{d}}^{2}}$$$$n=\frac{{\left(1.96\right)}^{2}\left(0.5\right)\left(0.5\right)}{(0.05)2}=384$$; where n is the required sample size, Z/2 = 1.96 (the Z score corresponds to a 95% confidence level), P is the proportion of patients with utility greater than the mean, and d is the margin of error, which was set at 0.05. A total of 422 patients were approached, with a 10% margin of error for inappropriate and non-responsive responses. To recruit participants, a systematic random sampling technique was used.

According to ambulatory dermatologic clinic records at the University of Gondar, 610 chronic patients with DDs have visited the clinic on average per month since chronic skin disease patients are advised to visit the dermatologic clinic for a minimum of one month and a maximum of three months. As a result, the total number of chronic skin disease patients visiting over three months was 1830. Given that the sample was collected within three months, the sampling fraction (k-interval) is 4.33 (approximately four). The subjects were selected randomly as per randomized sampling method for data collection.

### HRQoL outcome measures

HRQoL was the primary outcome of this study. The respondents’ HRQoL was assessed using a generic EQ-5D-5 L questionnaire with a 5-level response (from 1 = no problem to 5 = extreme problem) and the EQ-VAS scale, on which the patient marks the overall state of health as a number (0 = worst imaginable state of health, 100 = best imaginable state of health). While the utility value difference between the worst and best is 0–1 (death is 0 and perfect health is 1), the EQ-5D-5 L is highly-sensitive, easy to use, and can generate a single total score based on socially relevant HRQoL measures [20, 21]. Additionally, the EQ-5D-5 L instruments have a good psychometric property in terms of reduced ceiling effects, increase informativity, improved convergent and known-groups validity to measure the health outcome of patients with DDs [22, 23].

### Data collection techniques and instruments

Patients were interviewed face-to-face using Amharic questionnaires that included socio-demographic and EQ-5D-5 L scales. The socio-demographic section of the questionnaire inquiries about the respondent’s age, gender, residence, marital status, level of education, occupation, monthly income, health insurance, use of any alternative medication, medication availability, and affordability, and history of any substance use (alcohol, tobacco, or khat). Clinical characteristics were also collected by data collectors through medical chart review.

The Amharic version of the EuroQol Group’s Portable Valuation Technology (EQ-PVT) protocol was used to create the Amharic version of the EQ-5D-5 L. It was possible and culturally acceptable to estimate preferences for health states [24].

### Data quality control

The study questionnaire was meticulously designed to collect all the necessary information. Data collectors were trained prior to collecting data. A pretest was conducted by randomly selecting patients from their medication records to ensure the uniformity and understandability of the data collection tool, and any necessary changes were made. The pre-tested patients were excluded from the final analysis. Data was gathered by the investigator and trained nurses. In addition, the visual method was used to recheck checklist accuracy and completeness for any missing, incorrect, or unreadable information. Any discrepancies or ambiguities were quickly resolved.

### Data analysis and interpretation

Before coding, the data was checked for completeness and then entered into the Epi Info Version 7 database and exported to SPSS Version 26 for analysis. The study patients’ characteristics were described using descriptive statistics such as means, medians, proportions, tables, and figures. First, all statistical methods for all variables were checked to ensure that they met the test assumptions (normality test, correlation coefficients test, linearity test, outliers test, multicollinearity test, and homoscedasticity test), and those that did were included in the multivariable analysis (age, gender, residence, marital status, level of education, health insurance, use of alternative medication, alcohol use, khat chewing, duration of the disease, duration of the medication, treatment modality, and comorbidity). However, the following variables were excluded from the multivariable analysis due to one or more deviations from the linear regression assumption: occupation, monthly income, affordability, availability, smoking history, and type of skin diseases. The data’s linearity was investigated by plotting the dependent variable on the y-axis and the independent variables on the x-axis in separate scatter plots. To investigate the relationship between the mean HRQoL score and another continuous variable, the P-P plot was used. Similarly, by drawing a line through the center of each level’s observations, the linear relationship between categorical variables was investigated. The variables with no linear relationship to the outcome were removed from the analysis. The variation around the regression line was examined by plotting the standardized residuals vs. the standardized projected values of the dependent variable, and it was found to be constant for all xi values for each X variable. The data distribution was examined using a histogram, a normal probability map of the residuals, and the Shapiro-Wilk test, which revealed that the residuals were roughly normally distributed. The unstandardized coefficient was used to express the regression analysis results. The beta coefficient, expressed in standard deviation units, represents the average change in the dependent variable for each unit increase in the predictor variable. Bivariable and multivariable linear regression were used to identify the independent predictor variables of HRQoL. The fitness of the model was assessed, as well as the strength and direction of associations between the dependent and independent variables, using an adjusted odds ratio (AOR) and 95% confidence intervals (CI). To declare statistical significance, a p-value less than 0.05 was used. The models’ suitability was assessed using the goodness-of-fit test.

## Results

### Sociodemographic characteristics of participants

Total 422 dermatologic patients approached for this study, 400 (a response rate of 94.8%) were included in the final analysis. More than half of the participants (56%) were female. The participants’ mean (± SD) age was 39.8 (± 17.2) years. Higher number of the patients (58.3%) had health insurance. Half of the participants 193 (48.3%) had used traditional medications to treat their skin illnesses (Table 1).

**Table 1:** Sociodemographic characteristics of dermatology patients at the dermatologic clinic of UoGCSH, Ethiopia (N = 400)

Variables	Category	N (%)
Sex	Male	176 (44)
	Female	224 (56)
Age of participants	Mean (± SD)	39.79 (17.17)
	18–35	211 (52.8)
	36–60	130 (32.5)
	> 60	59 (14.8)
Residence	Urban	240 (60)
	Rural	160 (40)
Marital status	Single	124 (31)
	Married	221 (55.2)
	Divorced	15 (3.8)
	Widow	40 (10)
Educational status	Unable to read and write ^a^	89 (22.3)
	Primary (1–8)	93 (23.3)
	Secondary (9–12)	107 (26.8)
	College and above	111 (27.8)
Occupation	Farmer	90 (22.5)
	Government employee	71 (17.8)
	Business/self-employee	87 (21.8)
	Student	72 (18)
	Housewife	71(17.8)
	Unemployed	10 (2.5)
Monthly income (Birr)	≤ 860	147 (36.8)
	861–1500	77 (19.3)
	1501–3000	82 (20.5)
	3001–4999	67 (16.8)
	≥ 5000	27 (6.8)
Health insurance	Yes	233 (58.3)
	No	167 (41.7)
Alcohol drinking	No	167 (41.8)
	Yes	233 (58.2)
Khat chewing	Yes	28 (7)
	No	372 (93)
Cigarette smoking	No	339 (84.7)
	Yes	61 (15.3)
Medication availability	Yes	397 (99.2)
	No	3 (0.8)
Medication affordability	Yes	375 (93.2)
	No	25 (6.8)
Use of alternative medicine	Yes	193 (48.3)
	No	207 (51.7)

^a^ Unable to read and write; refers patients comes from illiterate population

### Clinical characteristics of patients with dermatological disorders

More than 64.2% of patients started to experience dermatologic symptoms before turning 40 years old. More than half (53%) of the patients had a disease duration of fewer than 5 years. Frequency of psoriasis was the highest at 13.5%, followed by acne (11.5%) and skin infections (11.2%). Around 136 (34%) patients were living with at least one kind of coexisting disease. The majority of the participants used the topical mode of treatment (273, 68.3%) (Table 2).

**Table 2:** Clinical characteristics of patients with dermatological disorders at the ambulatory dermatologic clinic of UOGCSH, Ethiopia 2022

Variable	Category	N (%)
Age at initial diagnosis	< 40 years	257 (64.2)
	≥ 40 years	143 (35.8)
Duration of disease	< 5 years	255 (63.7)
	≥ 5 years	147 (36.3)
Duration on medication	< 3 years	258 (64.5)
	≥ 3year	141 (35.5)
The type of skin disease	Psoriasis	50 (13.5)
	Acne	44 (11.5)
	Skin infection	43 (11.2)
	Eczema	42 (10)
	Dermatitis	39 (8.8)
	Keloid	31 (7.7)
	Leprosy	17 (4.3)
	Rosea	19 (4.8)
	Skin fungal infection	39 (8.8)
	Scabies	10 (2.5)
	Post-kala-azar cutaneous leishmaniasis	25 (6.3)
	Melasma	20 (5.2)
	Vitiligo	21 (5.4)
Comorbidity	Yes	136 (34)
	No	264 (66)
Treatment modality	Topical	273 (68.3)
	Systemic	88 (22)
	Both topical and systemic	39 (9.8)

### The severity of dermatologic diseases

**Fig. 1:**
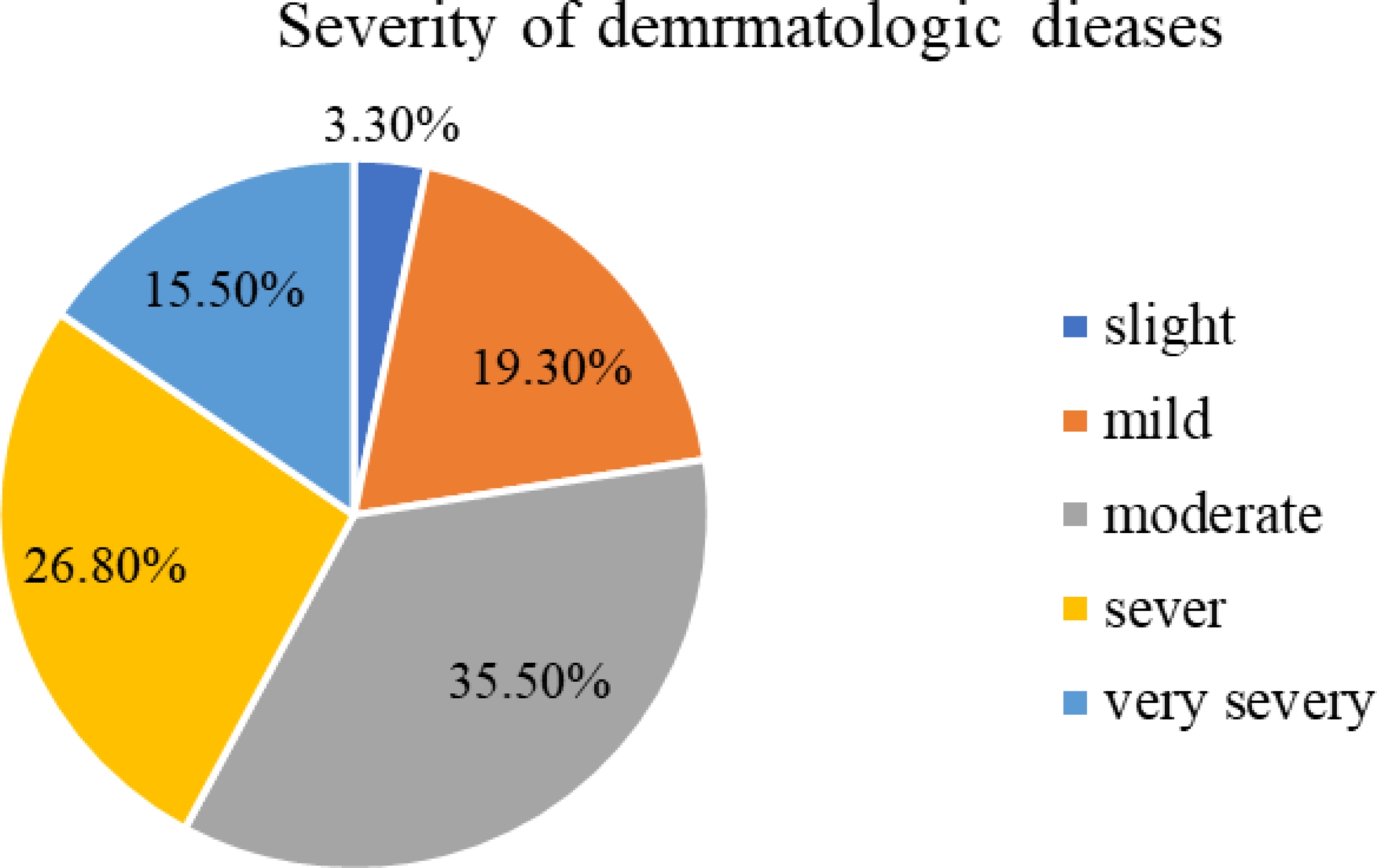
Severity of dermatological disorders of the participant

Regarding the severity of skin diseases, more than three-fourths (35.5%) of the participants had moderate skin diseases and only 3.3% of the participants were categorized as slight skin diseases according to physician diagnosis (Fig. 1).

### Prescribing pattern of medications at the dermatologic clinic

In this study, patients received topical corticosteroids for treating psoriasis. The most commonly prescribed steroids are Betamethasone dipropionate, Betamethasone dipropionate plus amphotericin B, and Mometasone furoate, with frequency of 62 (15.5%),41 (10.3%), and 86 (21.5%), respectively. Methotrexate was used by 18 (4.5%) participants only (Table 3).

**Table 3:** Prescribing pattern of medications at the dermatologic clinic of UOGCSH, Ethiopia 2022

Treatment modality	Total N (%)
**Topical corticosteroids**	
Betamethasone dipropionate	62 (15.5)
Betamethasone dipropionate plus amphotericin B	41 (10.3)
Betamethasone dipropionate plus ketoconazole	20 (5)
Betamethasone valerate	21 (5.3)
Clocortolone pirolate	35 (8.3)
Mometasone furoate	86 (21.5)
Clobetasone propionate	26 (6.5)
**Other topical agents**	
Salicylic acid	8 (2)
Fusidic acid	12 (3)
Tretinoin cream	71 (17.8)
**Systemic agent**	
Methotrexate	18 (4.5)
Total	400 (100%)

### HRQOL of patients with dermatological disorders

The overall mean (± SD) scores of EuroQol and EQ-VAS of the participants were 1.92 (0.74) and 68.93 (24.24), respectively. Concerning HRQoL domains, pain or discomfort was the most common health problem in patients with skin diseases (22.3%), followed by anxiety or depression (15.3% (Table 4).

**Table 4:** Proportion and mean score for each EQ-5D-5 L domain of the participant dermatologic clinic of UOGCSH, Ethiopia 2022

EQ-5D-5 L Domains	No problem	Slight problem	Moderate problem	Sever problem	Unable to do anything	Mean (± SD)
Mobility	285 (71.3)	49 (12.3)	35 (8.8)	26 (6.5)	5 (1.3)	1.54 (0.98)
Self-care	256 (64)	76 (19)	42 (10.5)	23 (5.8)	3 (0.8)	1.60 (0.94)
Usual activity	199 (49.8)	95 (23.8)	64 (16)	38 (9.5)	4 (1)	1.88 (1.05)
Pain /discomfort	46 (11.5)	126 (31.5)	129 (32.3)	89 (22.3)	19 (2.5)	2.73 (1.01)
Anxiety /depression	187 (46.8)	118 (29.5)	61 (15.3)	29 (7.2)	5 (1.3)	1.97 (1.00)
Overall EUROQOL-5D-L						1.92 (0.74)
VAS mean score						68.93 (24.24)

### Associations between the HRQoL and other variables

To identify potential variables influencing the health-related quality of life of patients with various skin diseases, a linear regression analysis was used. The linear regression model fitness was tested and found to be significantly associated (F = 13.45; p < 0.001). A multivariable analysis identified factors that may be associated with HRQOL, including age, treatment modality, the severity of the skin disease, and the presence of comorbidity. The regression results showed that the model explained 44.7% of the variance, and the variance inflated factor was less than five for all variables.

Respondents whose age increased by one increased the risk of severely impaired HRQoL scores by 0.011 times (95%CI (0.006, 0.016), p < 0.001). Compared with participants who had a combination of both topical and systemic dermatologic medications, those who had only topical preparations and those who took only systemic medications had decreased risks of severely impaired HRQoL, with a β score of -0.399 (95% CI: -0.602, -0.191), p < 0.001), and a β score of -0.378 (95% CI: -0.607, -0.149), p = 0.002, respectively. Patients having slight, mild, and moderate skin diseases had less likely risk of having impaired HRQoL compared to patients who had very severe skin diseases, with a β score of -0.654 (95% CI: -1.01, -0.290), p < 0.001; a score of -0.748 (95% CI: -0.960, -0.538), p < 0.001; and a score of -0.465 (95% CI: -0.642, -0.283), p < 0.001; respectively. Respondents who had a longer duration with dermatological conditions had 0.046 times (95%CI (0.015, 0.352), p = 0.013) the risk of severely impaired HRQoL. On average, patients who had other comorbid conditions other than skin diseases had 0.251 times (95% CI (0.096, 0.402; p = 0.002) more severely impaired HRQoL than those without comorbid conditions. The above-listed variables had been identified as significant predictors of health-related quality of life, but the other variables had lost their effect on multiple variable analyses (Table 5).

**Table 5:** Factors associated with poor quality of life among patients with Psoriasis attending the dermatologic clinic of UOGCSH, Ethiopia 2022

Variable		SLR β (95% CI)	p-value	Adj R^2^%	MLR β (95% CI)	p-value
Age		0.021 (0.017,0.025)	**< 0.001**	22.2	0.011(0.006,0.016)	**< 0.001**
Sex	Male	-0.119(-0.269,0.032)	**0.123**	0.03	0.018(-0.115,0.151)	0.789
	Female	0			0	
Residency	Urban	0.393 (0.244,0.541)	< 0.001	6.1	0.102(-0.047,0.251)	0.180
	Rural	0			0	
Marital status	Single	-0.762 (-1.02,-0.50)	**< 0.001**	9.3	0.074(-0.193,0.339)	0.584
	Married	-0.345(-0.592,-0.099)	0.006		0.035(-0.178,0.249)	0.748
	Divorced	-0.241(-0.672,0.192)	0.273		0.013(-0.357,0.381)	0.947
	Window	0			0	
Level of education	No formal education	0.750 (0.55,0.949)	< 0.001	12.5	0.086(-0.161,0.331)	0.494
	Primary (1–8)	0.508 (0.311,0.706)	**< 0.001**		0.154(-0.521,0.358)	0.145
	Secondary (9–12)	0.296 (0.106,0.486)	0.002		0.068(-109,0.245)	0.451
	College and above	0			0	
Health insurance	Yes	-0.378 (-0. 526, -0.235)	< 0.001	5.8	0.007(-0.134,0.151)	0.943
	No	0			0	
Alcohol use	Yes	0.280 (0.130,0.423)	**< 0.001**	3	0.068(-0.068,0.204)	0.321
	No	0			0	
Khat chewing	Habitual	0.72 (-0.221,0.366)	0.062	0.2	-0.009(-0.254,0.234)	0.940
	Not habitual	0			0	
duration since diagnosis	< 5 years	0.243 (0.094,0.402)	**0.002**	2.2	0.046(0.015,0.352)	**0.013**
	≥ 5 years	0			0	
Duration on medication	< 3 years	0.176 (0.019,0.332)	**0.028**	1.04	-1.08(-0.304,0.088)	0.273
	≥ 3 years	0			0	
Treatment modality	Topical	-0.663(-0.912, -0.416)	**< 0.001**	6.5	-0.399(-0.602, -0.191)	**< 0.001**
	Systemic	-0.450 (-0.721, -0.171)	0.002		-0.378(-0.607, -0.149)	**0.002**
	Both	0			0	
The severity of the diseases	Slight	-1.02 (-1.407, -0.633)	<0.001	24.6	-0.654(-1.01, -0.290)	**< 0.001**
	Mild	-1.08 (-1.310, -0.866)	< 0.001		-0.748(-0.960, -0.538)	**<0.001**
	Moderate	-0.68 (-0.879, -0.482)	**< 0.001**		-0.465(-0.642, -0.283)	**<0.001**
	Sever	-0.212 (-0.420, -0.004)	0.045		-0.103(-0.292,0.086)	0.289
	Very severe	0				
Use of traditional medicine	Yes	0.279 (0.131,0.426)	< 0.001	3.1	-0.091(-0.413,0.112)	0.775
	No	0			0	
Comorbidity	Yes	0.671 (0.528, 0.812)	< 0.001	17.6	0.251(0.096, 0.402)	**0.002**
	No	0			0	

SLR: simple linear regression MLR: multiple linear regressions; CI, Confidence interval, adjusted R^2^ =44.7 ,F = 13.45; P < 0.001,VIF < 5; bold figures indicate p < 0.05

## Discussion

To the best of our knowledge, this is the largest cross-sectional study to look HRQoL and factors influencing a standardized HRQoL among patients with skin diseases, with the potential to account for multiple factors for dermatological disorders. The five independent factors identified (age, severity, duration, treatment modality, and comorbidity) were significantly related to HRQoL, as measured by EuroQol domain scores.

The EuroQol tool was used to assess HRQoL among patients with various DDs in this institution-based study. According to the findings of this study, the overall mean (± SD) HRQoL and VAS scores were 1.92 (± 0.74) and 68.93 (± 24.24), respectively, out of 5 and 100. Our participants’ overall mean score was higher than the findings obtained during a study at Vietnam [25]. This could be because majority of our study population was elderly, and there was significant socioeconomic difference between the study groups.

In terms of HRQoL domains, pain or discomfort and anxiety or depression were the two most significantly affected parameters. The fact that most DDs symptoms are visible, as well as the impact that emotional anguish has on daily activities and employment, may have been the most visible consequence. Our findings reaffirmed the importance of considering the psychological impairment of patients withDDs. This is consistent with previous research findings [9, 12, 26–29]. Hence the link between DDs and mental problems are related, psycho dermatology is categorized into three mechanisms: The primary psychological diseases that cause self-inflicted diseases of the skin (trichotillomania); secondary psychophysiological disorders brought on by skin conditions that cause different emotional states (stress). Third, psychiatric disorders brought on by disfiguring skin (ichthyosis, acne conglobata, vitiligo), which can result in states of fear, depression, or suicidal thoughts [30].

Dermatological disorders have a negative impact on patients’ mental and physical health [31–33], which can greatly decline HRQoL [32, 33]. Patients may experience sadness or anxiety circumstances that can be explained by the advent of immune problems and elevated in proinflammatory cytokine concentrations [25]. In addition to the painful or itchy conditions that patients must endure throughout the course of diseases [34, 35]. Furthermore, previous research has suggested that people with skin conditions may face social stigma as a result of their atypical skin [36, 37]. Anxiety and depression were reported by more than 70% of the participants in this study, which was higher than the prevalence of these conditions in populations with other chronic illnesses like respiratory and cardiovascular diseases [32, 36].

As expected, the severity of the illness had an effect on the HRQoL of patients with DDs. Similar findings have been reported in studies among patients with psoriasis, acne, cutaneous lupus erythematosus (CLE), and alopecia areata [9, 10, 14, 27]. The current findings also revealed a link between significantly lower HRQoL scores and disease duration, as well as other chronic diseases. This demonstrates the chronic nature of skin diseases and their long-term consequences which was also found previously among patients suffering with psoriasis [14, 26] and CLE [10]. However, no statistically significant relationships have been discovered in studies on vitiligo [11], urticaria [12], and alopecia areata [27].

The participants’ age was found to be an independent predictor of HRQoL in dermatologic patients. This finding is consistent with the findings from Portugal [38]. The cause could be age-related increases in the risk of comorbidities and physical activity limitations, which lead to poorer HRQoL. Dermatological disorder patients who received single therapy had better HRQoL than those who received combination therapy. These finding are consistent with the study conducted in Portugal they disclosed that patient those who have received single treatment had better HRQoL than those received multiple treatment [38]. This finding may be explained by the fact that single therapy has higher treatment satisfaction, fewer pills or injections, lower medication costs, and potentially fewer side effects than combination therapy.

### Strengths and limitations of this study

The results of this study could be used to make several recommendations. First, patients with skin illnesses should receive appropriate pain treatment and psychological counseling services, given that anxiety, depression, and pain are extremely common in this population. In addition, using a brief, straightforward tool, regular monitoring of HRQoL should be carried out to monitor the development in addressing the disparities between various groups of people, such as socioeconomic groups of people with skin diseases.

One of the study’s strengths is that it included a large number of people with skin conditions and adjusted for a total of 12 skin conditions before identifying characteristics linked to severely reduced HRQoL. By controlling for other variables, these findings can be used to assess the likelihood of severely reduced HRQoL in patients with the common skin illnesses studied in during this study.

This study has a few methodological issues that must be addressed. First, due to the limitations of the cross-sectional design, we were unable to determine the precise causal links between predictor variables and HRQoL. As a result, more longitudinal research is required. Second, measurement error was possible because the HRQoL data were self-reported subjectively.

## Conclusions

Patients with DDs were found to have compromised HRQoL. Pain/discomfort and anxiety/ depression are the two most severely affected domain of HRQoL compared to the other domain. Socio- demographic, clinical and medication related variables were significantly associated with HRQoL of DDs patients. Therefore, future researchers would be worked on HRQoL and its associated factors in patients with DDs on at lower health care facilities that may not have the infrastructure and resources available in higher level facilities.

## Data Availability

The datasets generated and/or analyzed during the current study are not publicly available due to data security regulations and participant confidentiality, but are available from the corresponding author on reasonable request.

## Abbreviations

DDs: Dermatological diseases
CLE: Cutaneous lupus erythematosus
EQ-5D-5L: The EuroQol-5 Dimensions-5 Levels
HRQoL: Health related quality of life
QoL: Quality of life
UoGCSH: University of Gondar Comprehensive specialized hospital
